# Pesticides and Parabens Contaminating Aquatic Environment: Acute and Sub-Chronic Toxicity towards Early-Life Stages of Freshwater Fish and Amphibians

**DOI:** 10.3390/toxics11040333

**Published:** 2023-03-31

**Authors:** Denisa Medkova, Aneta Hollerova, Barbora Riesova, Jana Blahova, Nikola Hodkovicova, Petr Marsalek, Veronika Doubkova, Zuzana Weiserova, Jan Mares, Martin Faldyna, Frantisek Tichy, Zdenka Svobodova, Pavla Lakdawala

**Affiliations:** 1 Department of Animal Protection and Welfare & Veterinary Public Health, Faculty of Veterinary Hygiene and Ecology, University of Veterinary Sciences Brno, 612 42 Brno, Czech Republic; 2 Department of Zoology, Fisheries, Hydrobiology and Apiculture, Faculty of Agrisciences, Mendel University in Brno, 613 00 Brno, Czech Republic; 3 Department of Animal Breeding, Animal Nutrition and Biochemistry, Faculty of Veterinary Hygiene and Ecology, University of Veterinary Sciences Brno, 612 42 Brno, Czech Republic; 4 Department of Infectious Diseases and Preventive Medicine, Veterinary Research Institute, 621 00 Brno, Czech Republic; 5 Department of Anatomy, Histology and Embryology, Faculty of Veterinary medicine, University of Veterinary Sciences Brno, 612 42 Brno, Czech Republic

**Keywords:** metazachlor, prochloraz, MCPA, methylparaben, butylparaben, propylparaben

## Abstract

Pesticides and personal care products are two very important groups of contaminants posing a threat to the aquatic environment and the organisms living in it.. Therefore, this study aimed to describe the effects of widely used pesticides and parabens on aquatic non-target biota such as fish (using model organisms *Danio rerio* and *Cyprinus carpio*) and amphibians (using model organism *Xenopus laevis*) using a wide range of endpoints. The first part of the experiment was focused on the embryonal toxicity of three widely used pesticides (metazachlor, prochloraz, and 4-chloro-2-methyl phenoxy acetic acid) and three parabens (methylparaben, propylparaben, and butylparaben) with *D. rerio*, *C. carpio*, and *X. laevis* embryos. An emphasis was placed on using mostly sub-lethal concentrations that are partially relevant to the environmental concentrations of the substances studied. In the second part of the study, an embryo-larval toxicity test with *C. carpio* was carried out with prochloraz using concentrations 0.1, 1, 10, 100, and 1000 µg/L. The results of both parts of the study show that even the low, environmentally relevant concentrations of the chemicals tested are often able to affect the expression of genes that play either a prominent role in detoxification and sex hormone production or indicate cell stress or, in case of prochloraz, to induce genotoxicity.

## 1. Introduction

Pesticides as well as personal care products are emerging contaminants that are becoming increasingly abundant in aquatic environments, with the ability to affect biota.

Over time, the global production of pesticides increased from 0.2 million tons in the 1950s to 5 million tons in 2000, and 4.1 million tons were applied in 2016 [1,2,3]. Their residues enter the aquatic environment through spray drift and runoff with rainwater from rainfall or irrigation [4]. Various properties such as their persistence and their bioaccumulation potential can have a negative effect on aquatic biota, fish, and other non-target organisms [1,2,3].

Metazachlor (MTCH) is a herbicide used in oil seed crops to control annual grass and broad-leaved weeds [5]. In the EU, MTCH is an approved active substance with authorization approval expiring by the end of July 2023 (may be prolonged) [6]. Due to its frequent use, residues have been found in surface waters as well as animal tissues. Mohr et al. [7] reported the concentrations of MTCH in surface waters up to 100 μg/L in European countries. A more recent study [8] reported the highest concentration in surface water for metazachlor oxanilic acid, a metabolite of MTCH, to be 519.48 ± 56.52 ng/L. Quintana et al. [9] reported MTCH concentrations in the range of 0.1–0.5 μg/L in the low estuary of the Llobregat River (Barcelona, Spain). Concerning the concentrations reported in fish tissues, values from 1.1 ng/g of MTCH [8] up to 300 ng/g [10] were measured. Lazartigues et al. [10] established a biomagnification factor for European perch (*Perca fluviatilis*) and carp (*C. carpio*), in particular 3.7 ± 0.6 and 2.72 ± 0.1, respectively. Various studies have shown that exposure to MTCH results in negative effects on non-target organisms living in the aquatic environment [11,12].

Prochloraz (PCZ) is an imidazole fungicide that disrupts ergosterol synthesis by inhibiting cytochrome P450 (CYP) 14α-demethylase (CYP51) through an interaction with iron in the heme cofactor, effectively weakening fungal cell membranes [13]. In the EU, the approval of the use of this active substance expired by the end of the year 2021 [6]. However, PCZ is frequently detected in various environmental matrices such as water, sediment, and soil. The frequently detected concentrations of PCZ in stream water within an agricultural catchment in southern Sweden range from trace concentrations from 0 to 2 μg/L [14]. Campos-Mañas et al. [15] measured PCZ in Spanish wastewater with an average concentration of 14 ± 4 ng/L. In Mediterranean rivers, PCZ concentrations were 26.5 ng/L in the influent and 22.2 ng/L in the effluent of a wastewater treatment plant (WWTP), which suggests a 16.4% removal rate of PCZ in WWTP [16]. Concerning PCZ concentrations in tissues, [17] reported concentrations up to 82.79 μg/kg in fish muscle tissues coming from the Spanish river Júcar. Concentrations up to 37.8 μg/kg were measured in fish muscle in fish found in the Douro estuary (northeast Atlantic) [18]. Dalhoff et al. [19] estimated the bioaccumulation factor to be 15.6 L/kg (study in *Daphnia magna*). Known toxic effects on the non-target aquatic animal are described and discussed in Section 3.

The 4-chloro-2-methyl phenoxy acetic acid (MCPA) is a selective herbicide with systemic and hormonal action for the control of broadleaf weeds in cereals and other crops [20]. In the EU, MCPA is an approved active substance with the authorization approval expiring by the end of October 2023 (may be prolonged) [6]. Its residues have been found in water bodies worldwide. For example, in the northeast of France, surface water concentrations of MCPA were found to be 26.5 μg/L [21]. Morton et al. [22] reported MCPA concentrations in Irish rivers to be greater than 4.33 μg/L. Barbieri et al. [23] found that the MCPA concentrations in the Ebro River Delta (Spain) ranged from 0.1 to 8.2 μg/L, with an average value of 1.7 μg/L. Regarding the concentrations of MCPA in tissues of aquatic non-target biota, Tyohemba et al. [24] reported the MCPA content in the muscle tissues of two species of fish found in South Africa (Lake St Lucia), namely the North African catfish (*Clarias gariepinus*) and Mozambique tilapia (*Oreochromis mossambicus*), to be 18.8 ± 12 and 14.3 ± 14 ng/g, respectively. Bermúdez-Saldaña et al. [25] estimated the bioconcentration factor of MCPA for fish to be 0.5. MCPA represents a potential risk to wildlife and aquatic ecosystems [26].

Together with pesticides, parabens are a major public health issue connected with their excessive use and, consequently, negative impact on human and animal health and the environment [27]. Parabens are widely applicated in food, pharmaceuticals, and personal care products [28,29]. Parabens have the potential to bioaccumulate and can be toxic to aquatic species. As endocrine-disrupting compounds, parabens can competitively bind to estrogen receptors, alter the production and degradation of endogenous steroids, and influence steroid-sensitive tissues, in turn impacting the nervous system, immune system, and lipid homeostasis [30]. Unfortunately, the traditional methods used in WWTPs, namely the adsorption process, activated sludge, and advanced oxidation processes, are not effective in removing these types of contaminants [31]. Fortunately, at least in the EU, studies show that the use of parabens has a decreasing trend combining both the EU regulation as well as customer preference to use paraben-free products [32]. Methylparaben (MTP) and propylparaben (PPP) are the most used parabens and are present in products together [33].

MTP is the ester of p-hydroxybenzoic acid used as an antimicrobial preservative in cosmetics, drugs, and food [33]. It is the most detected paraben in the environment [34]. Hu et al. [34] reported the concentration of MTP in the surface water of Nigeria to reach 527 µg/L and 212 µg/L in groundwater. In China, MTP was reported to have the highest concentration of 3142 ng/L [35]. Pollution levels at 1062 and 3173.9 ng/L have been reported for MTP in the Pearl River Delta and Xiangjiang River of China, respectively. In Europe, up to 10.1 μg/L of MTP was previously detected in landfill leachate in Poland. Hu et al. [36] showed in their study that MTP shows a predominant existence in the majority of the analyzed tissues, where the highest concentration was recorded to be 865 ng/g wet weight in the livers of bottlenose dolphins in the United States. The bioconcentration factor of MTP is 2.5–137.3 in marine mussels (*Mytilus galloprovincialis*) [37].

MTP was found to impact neural functions in zebrafish. However, research still lacks a comprehensive understanding of its neurotoxicity [34]. Raja et al. [38] observed anxiety-like behavior in treated *D. rerio* larvae and decreased exploratory behavior after exposure to 0.1 µg/L and 1 µg/L of MTP. Hu et al. [34] carried out trials on zebrafish exposed to concentrations of 1, 3, and 10 μg/L of MTP for 28 days. This sub-chronic exposure caused, even at an environmentally realistic concentration of 10 μg/L, remarkable perturbation of brain proteome structure in both male and female zebrafish. Furthermore, degenerative vacuolization of hepatocytes was prevalent in female livers, which was characterized by the rupture of the cell membranes and small nuclei.

PPP, an n-propyl ester of p-hydroxybenzoic acid, is both a naturally occurring as well as industrially produced substance that is used as a preservative in foods, cosmetics, toiletries, and pharmaceutical formulations [39]. PPP has bactericidal and fungicidal effect. It was assessed as an estrogenic and endocrine disruptor [40]. Calma et al. [41] detected PPP in wastewater, freshwater systems, and even bottled drinking water at concentrations reaching 20 µg/L, 3.1 µg/L, and 0.023 µg/L, respectively. Wang et al. [42] reported PPP concentrations in fish muscle tissue coming from China’s Taihu Lake to be between 261 and 1710 pg/g. The PPP bioaccumulation factor was estimated at 2.59–3.57 in wild fish from the Yangtze River (China) [43].

Butylparaben (BTP), a butyl p-hydroxybenzoate, is the most potent among alkyl paraben and has the greatest estrogenic effects [44]. Okoye et al. [45] detected BTP at concentrations of 0.0064–0.071 μg/L in Northeast Africa—Egypt. Bolujoko et al. [46] reported BTP concentrations between 1.9–11.0 μg/L in the São Paulo Rivers in Brazil. In Chile, Becerra-Herrera [47] measured BTP concentrations between 0.4–0.9 μg/L. In muscle tissues, BTP was reported in fish (Crucian carp, bighead carp, silver carp, topmouth culter, and large icefish) from China (in Taihu Lake) in concentrations from 261 to 1710 pg/g [42]. Bioaccumulation factor of 2.3–3.3 of BTP was estimated in wild fish from the Yangtze River (China) in the study of Yao et al. [43].

This study aimed to describe the effects of widely used pesticides and parabens on aquatic non-target biota such as fish (using model organisms *Danio rerio* and *Cyprinus carpio*) and amphibians (using model organism *Xenopus laevis*) using a wide range of endpoints in order to see whether these pollutants can have a negative effect on early-life stages of model aquatic organisms. An emphasis was placed on using sub-lethal concentrations that are partially relevant to the environmental concentrations of the substances studied. Based on the data from the literature, PCZ acute toxicity seems to be the highest regarding the chemicals selected for this study. Therefore, a sub-chronic toxicity test on the early-life stages of fish was added for PCZ in order to also describe the sub-chronic effects of sublethal concentrations of this compound.

## 2. Materials and Methods

### 2.1. Experimental Design

**Scheme 1:**
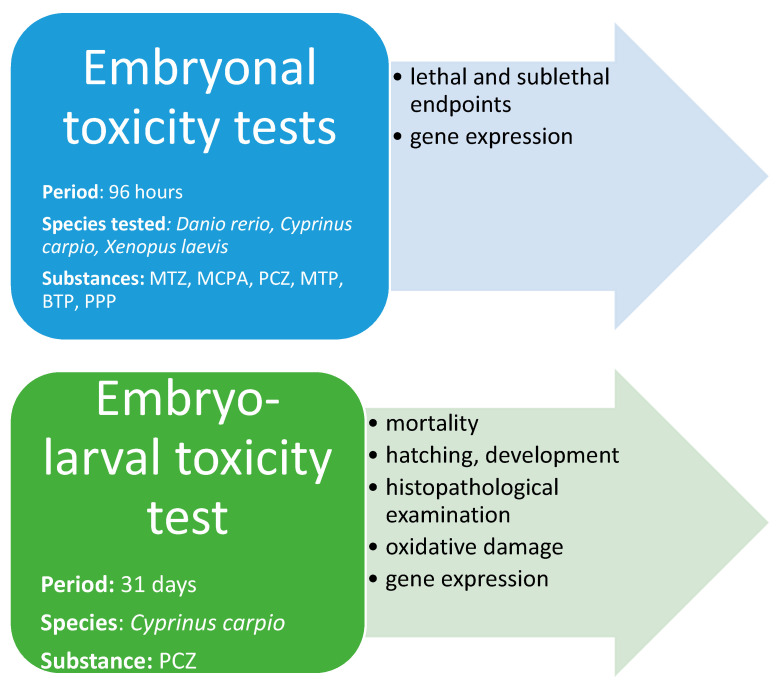
Tests carried out with the specification of the length of the test, species used, and substances tested.

The first part of the experiment was focused on the embryonal toxicity of three widely used pesticides (MTZ, PCZ, MCPA) and three parabens (MTP, BTP, PPP) with D. rerio, C. carpio, and X. laevis embryos. In the second part of the study, an embryo-larval toxicity test with C. carpio was carried out with PCZ. Since an extensive array of testing with three species, two different life-stages, and various contaminants was performed, a generalized flow chart (Scheme 1) describing how each species and life stage was presented below.

#### 2.1.1. Embryonal Toxicity Tests—Lethal and Sublethal Endpoints

Embryonic acute toxicity tests inspired by the Guideline for Test No.236: Fish Embryo Acute Toxicity (FET) [48] were carried out on zebrafish (*Danio rerio*), common carp (*Cyprinus carpio*) and African clawed frog (*Xenopus laevis*). *C. carpio* eggs were obtained from commercial farm Rybnikarstvi Pohorelice a.s. (Czech Republic), *D. rerio* eggs were obtained from the certified breeding of Mendel University in Brno (Czech Republic), and *X. laevis* eggs were obtained from the certified breeding of Masaryk University Brno (Czech Republic). Fertilized and well-developing eggs of each species were distributed in 24-microwell plates (TPP, Switzerland), while each plate represented one tested group (i.e., 24 embryos were tested in each group). The chemicals and the concentrations they were tested at are indicated in Table 1. All chemicals were purchased from Sigma-Aldrich (Prague, Czech Republic). The lowest tested concentrations were set up concerning environmentally relevant concentrations. Higher concentrations were selected to see if the response of the organism was concentration-dependent. For *D. rerio* and *C. carpio*, dilution water was prepared according to ISO 7346 [49]. For *X. laevis*, Marc’s modified ringer’s (MMR) [50] was used.

Groups and concentrations indicated in bold were also used in the study on gene expression assessment described in Section 2.1.2.

Tested eggs placed in microwell plates were exposed to 2 mL of testing solutions. Testing solutions were changed every 24 h to ensure that the real tested concentrations did not fall under 80% of their nominal values. Control groups were exposed to dilution media only. To exclude the toxic effects of the solvents ethanol and DMSO used in the test, control groups with the addition of 0.01% of ethanol and DMSO were also set up. Furthermore, the use of DMSO has been proven not to be toxic to fish in other scientific studies [51]. During the tests, the experimental animals were kept under controlled temperature (26 °C for *D. rerio* and 23 °C for *C. carpio* and *X. laevis*) and photoperiod (12 h light/12 h dark).

Every 24 h, screening to report mortality, hatching rate, and developmental malformations rate was carried out using the stereomicroscope (Leica, Weltzlar, Germany). The test ended at 96 h post fertilization (hpf).

#### 2.1.2. Embryonal Toxicity Tests—Study on Gene Expression

In this part of the experiment, fertilized eggs of *D. rerio*, *C. carpio*, and *X. laevis* were distributed into 6-well microplates (TPP, Switzerland) (15 embryos per well, 180 embryos per group in total). Groups from Table 1 were tested in concentrations indicated in bold (two concentrations in a group) together with the control group and control groups with the addition of solvents. The length of exposure and other conditions were the same as described in Section 2.1.1.

At 96 hpf, samples were taken as follows: eight replicates were created out of each tested group. Each replicate contained 10 mg of embryos from that particular testing group, which were transferred in 1.5 mL Eppendorf tubes. These samples were immediately fixed in RNAlater (Fisher Scientific, Pardubice, Czech Republic) for 24 h at 4 °C and subsequently stored at −80 °C until ribonucleic acid (RNA) extraction.

#### 2.1.3. Embryo-Larval Toxicity Test

To keep the number of experimental animals used in toxicity tests minimal, only PCZ was tested in this part of the study. Moreover, as indicated in the introduction part of this manuscript, PCZ seemed to be a chemical with high acute toxicity. Therefore, it was necessary to also reveal the prolonged effects of sublethal concentrations. The experimental design of this study was fairly similar to the one described in another study [52], and the test was conducted according to the Guideline for Test No. 210: Fish, Early-life Stage Toxicity Test [53]. Briefly, freshly fertilized eggs of *C. carpio* (of the same origin as described in Section 2.1.1) were placed into 1 L crystallization dishes with 100 eggs in each dish. PCZ was tested at concentrations of 0.1, 1, 10, 100, and 1000 µg/L. Control and control with the addition of 0.01% DMSO were also set up. The experiment was carried out in triplicate, meaning = that each tested group was formed of three dishes (300 eggs in total). Every 12 h, testing solutions in all the dishes were replaced, water quality was tested (pH, temperature, dissolved oxygen, nitrates, nitrites, and total ammonia), and mortality and hatching were noted. After consuming the yolk sac, the larvae were fed ad libitum using freshly hatched *Artemia salina* twice a day. The whole experiment was completed at 31 days post fertilization (dpf). Fish were killed with an overdose of an anesthetic tricaine methanesulfonate (MS 222, Sigma-Aldrich, Prague, Czech Republic), and the specimens were handled and stored as indicated in the sections below.

During and at the end of the test (in particular dpf 6, 13, 20, 27, and 31), 10 specimens from each group were sampled to determine developmental stages and morphological and condition characteristics. The samples were preserved in 4% formaldehyde. Developmental stages were estimated according to [54]. Morphological and condition characteristics were investigated according to our previous study [52]. At 31 dpf, 10 fish from each group were sampled for histopathological examination, 8 fish from each group were sampled for quantitative polymerase chain reaction (qPCR) (described further in Section 2.4), and the rest of the surviving fish (equivalent to at least 300 mg in each group) were used for the indices related to oxidative stress.

Samples dedicated to histopathological examination were stored in 10% formaldehyde. Samples for qPCR were immediately fixed in RNAlater (Fisher Scientific, Pardubice, Czech Republic) for 24 h at 4 °C and subsequently stored at −80 °C until ribonucleic acid (RNA) extraction. Samples taken to evaluate changes in antioxidant and biotransformation enzyme activity (superoxide dismutase (SOD), catalase (CAT), and glutathione-S-transferase (GST)), lipid peroxidation (TBARS), and oxidative damages to proteins and DNA were frozen immediately and stored at –80 °C until analysis.

Both histopathological examination and examination aimed at changes caused by oxidative stress were carried out by our previous study [52].

SOD activity was assessed using a commercial kit (Cayman Pharma, Neratovice, Czech Republic) as well as oxidative DNA damage (Cayman Pharmal, Neratovice, Czech Republic). The level of DNA damage was expressed as a concentration of 8-hydroxy-2’-deoxyguanosine (8-OHdG). Both methods are briefly described in a previous study by [55]. Similarly, oxidative damage to proteins was also determined using a commercial kit (Sigma-Aldrich, Prague, Czech Republic).

CAT, GST, and TBARS were analyzed by methods described by [52,56]. All indices were determined spectrophotometrically using Varioskan Flash spectral scanning multimode reader (Thermo Fisher Scientific, Waltham, MA, USA).

### 2.2. Assays on Gene Expression

The analysis of gene expression, in particular steps covering RNA extraction and reverse transcription and quantitative real-time polymerase chain reaction, was performed by the method described in detail by Hodkovicova et al. [57].

Sequences for primers outpointing the detoxification processes, sex hormone production, cell stress, and genotoxicity were taken from the literature or designed using the NCBI primer-blast design tool available online at https://ncbi.nlm.nih.gov/tools/primer-blast/, accessed on 30 June 2022, as specified in Table S1. Gene expression was related to the reference gene, i.e., elongation factor (elof or ef1α, with respect to model organism), which was selected as the optimal reference gene for data normalization using the NormFinder software (Moma, Aarhus, Denmark).

### 2.3. Control of the Tested Substances

#### 2.3.1. Verification of Real Concentrations of Pesticides in Testing Solutions

A sample of water (10 mL) was spiked with 100 μL of internal standard solution (2 mg/L in deionized water), filtered through a 0.7 µm glass filter (Pall Corporation, New York, NY, USA), and used for LC/MS analysis. A Thermo Scientific UHPLC Accela 1250 system was connected to a Thermo Scientific TSQ Quantum Access MAX Triple Quadrupole Instrument (Thermo Scientific, Waltham, MA, USA) equipped with a heated electrospray ionization probe. A Kinetex C18 (2.1 mm × 100 mm, 1.7 μm; Phenomenex, Torrance, CA, USA) column was used at a constant flow rate of 250 μL/min. The mobile phase consisted of a 0.1% water solution of formic acid (solvent A) and acetonitrile (solvent B). The gradient used was as follows: 0–3.0 min linear gradient from 20 to 80% B; 3.0–5.4 min held at 80% B; 5.4–6.3 min from 80 to 20% B; and 6.3–7.0 min held at 20% B for the column to re-equilibrate before the next injection. The full-loop injection volume of the sample was set at 2 μL. The heated electrospray ionization was operated in the positive mode under the following conditions: capillary temperature, 350 °C; vaporizer temperature, 350 °C; sheath gas pressure, 35 psi; auxiliary (drying) gas, 10 au; and spray voltage, 3 300 V. For our quality assurance and quality control program, the instrument was calibrated daily with multi-level calibration curves. Procedural blank and solvent blank were analyzed for every set of 10 samples. The inter-day precision expressed as a relative standard deviation was 10.3% for PCZ, 10.6% for MTCH, and 11.8% for MCPA. The limit of detection determined as a 3:1 signal versus noise value was 0.073 μg/L for PCZ, 0.066 μg/L for MTZ, and 0.91 μg/L for MCPA. Standards of PCZ, MTCH, and MCPA were purchased from Sigma-Aldrich (Prague, Czech Republic). Acetonitrile was purchased from Chromservis (Prague, Czech Republic) and showed LC/MS purity (≥99.9%). The analysis proved that the real measured concentration did not fall under 80% of their nominal values.

#### 2.3.2. Verification of Real Concentrations of Parabens in Testing Solutions

A sample of water (10 mL) was spiked with 100 μL of internal standard solution (2 mg/L in deionized water) and filtered through a 0.7 µm glass filter (Pall Corporation, New York, NY, USA) and used for LC/MS analysis. A Thermo Scientific UHPLC Accela 1250 system was connected to a Thermo Scientific TSQ Quantum Access MAX Triple Quadrupole Instrument (Thermo Scientific, Waltham, MA, USA) equipped with an atmospheric pressure chemical ionization probe. A Kinetex C18 (2.1 mm × 100 mm, 1.7 μm; Phenomenex, Phenomenex, CA, USA) column was used at a constant flow rate of 250 μL/min. The mobile phase consisted of a 0.1% water solution of formic acid (solvent A) and acetonitrile (solvent B). The gradient used was as follows: 0–3.0 min linear gradient from 40 to 90% B; 3.0–4.0 min held at 90% B; 4.0–4.4 min from 90 to 40% B; and 4.4–5.0 min held at 40% B for the column to re-equilibrate before the next injection. The full-loop injection volume of the sample was set at 2 μL. The atmospheric pressure chemical ionization was operated in the negative mode under the following conditions: capillary temperature, 325.0 °C; vaporizer temperature, 300.0 °C; sheath gas pressure, 35.0 psi; auxiliary (drying) gas, 10 a.u.; discharge current, 4.0 μA. For our quality assurance and quality control program, the instrument was calibrated daily with multi-level calibration curves. Procedural blank and solvent blank were analyzed for every set of 10 samples. The inter-day precision expressed as a relative standard deviation was 12.6% for MTP, 11.7% for PPP, and 11.6% for BTP. The limit of detection determined as a 3:1 signal versus noise value was 3.35 μg/L for MTP, 1.21 μg/L for PPP, and 1.11 μg/L for BTP.

Standards of MTP, PPB, and BTB were purchased from Sigma-Aldrich (Prague, Czech Republic). Acetonitrile from was purchased Chromservis (Prague, Czech Republic) and showed LC/MS purity (≥99.9%). The analysis proved that the real measured concentration did not fall under 80% of their nominal values.

### 2.4. Statistical Analysis

Statistical analysis was conducted using Unistat 5.6 (Unistat Ltd., London, United Kingdom). Cumulative mortality, developmental stages, early ontogeny, and hatching data were analyzed using chi-square and contingency tables.

Regarding oxidative stress indices and mRNA expression, all data were tested for normal distribution by the Shapiro–Wilk test, and homogeneity of variance was tested using the Bartlett test. When the normality of parameters was achieved, parameters were assessed with a one-way ANOVA test and Dunnett’s multiple comparison test. Exposed groups were compared with the control group with a statistically significant difference if *p* < 0.05 and a highly statistically significant difference if *p* < 0.01. Box plot graphs were made by using median, Q1, and Q2 with whiskers of maximum 1.5 interquartile range and outliners denoted as points. No statistically significant difference was found between the control and control with the addition of solvents. Therefore, all figures just show one control that represents all the control groups in the tests.

## 3. Results and Discussion

### 3.1. Embryonal Toxicity Tests—Lethal and Sublethal Endpoints

#### 3.1.1. Effects of Pesticides

During these 96 h of tests, mortality, the occurrence of malformations, and hatching rates of *D. rerio*, *C. carpio*, and *X. laevis* embryos after exposure to MTCH, PCZ, and MCPA were observed.

Zebrafish (*D. rerio*)

At 24 hpf, a significantly higher malformation rate (undeveloped eyes) was reported at the highest tested concentration of 100,000 µg/L MTCH (*p* < 0.001). Accordingly, at 48 hpf, 100% mortality was observed in this group. Neither increased mortality nor increased malformation rate was observed in the other groups exposed to MTCH.

Exposure to PCZ did not cause any significant effect on malformation rate, hatching, or mortality, with only one exception of 100% mortality observed at 24 hpf in the highest-exposed group of 20,000 µg/L.

MCPA did not have any significant effect on the mortality *of D. rerio* embryos even in the highest tested concentration of 100,000 µg/L.

Regarding the occurrence of malformations in our study, exposure to MCPA at 100,000 µg/L resulted in an increased rate of malformations (heart edemas, blood clots) after 72 hpf (70.8% of specimens, *p* < 0.01). Additionally, delayed hatching was observed during exposure in groups exposed to 500, 5000, and 100,000 µg/L (*p* < 0.01).

Common carp (*C. carpio*)

Similarly, MTCH caused 100% mortality of *C. carpio* embryos in the highest tested concentration (100,000 µg/L) (*p* < 0.01). The concentrations of 500 and 5000 µg/L of MTCH significantly prolonged the time of hatching in comparison to other groups and the control (*p* < 0.05).

PCZ caused 100% mortality in the highest tested concentration (20,000 µg/L) (*p* < 0.01) at 72 hpf. All the embryos from this group developed heart edemas as well as tail-spine deformities before their death. At the concentration of 200 µg/L, the hatching rate at 72 hpf was lower in comparison to the control (*p* < 0.01).

Again, similarly to *D. rerio*, MCPA had no statistically significant effect on the mortality of common carp embryos but had some effects on hatching and malformation rates. Malformations such as heart edemas and blood clots were observed at the highest tested concentration after 96 hpf (29.1% specimen, *p* < 0.01). The hatching rates were lower (*p* < 0.01) at the concentrations of 50 and 500 µg/L at 72 hpf in comparison to the control.

African clawed frog (*X. laevis*)

MTCH did not have any negative effects on hatching and malformation rates. However, the exposure to concentrations of 5000 and 100,000 µg/L resulted in significantly higher mortality (*p* < 0.01), in particular 45.8% and 100%, respectively.

Exposure to PCZ resulted in significantly higher mortality in groups exposed to 200 and 20,000 µg/L (*p* < 0.01), in particular 70.8 and 100%, respectively.

No significant effects on either malformation or hatching rates were observed.

Regarding MCPA, only the highest tested concentration of 100,000 µg/L resulted in significantly increased mortality (100%). No other effects were noted.

In the scientific literature, MTCH is reported to be moderately toxic to fish. The 96hLC_50_ value of MTCH determined for *C. carpio* is 15 mg/L [58]. Therefore, the 100% mortality in the highest tested groups of MTCH among the species in our study is understandable. However, it has been reported that MTCH has pronounced effects on aquatic macrophytes and aquatic ecosystem function at concentrations exceeding 5 µg/L [11,59].

The 96hLC_50_ value for *D. rerio* was estimated to be 1.5 mg/L of PCZ in a study carried out by Guo et al. [60] and 8.5 mg/L for embryos and 4.6 mg/L for adults in a study published by Domingues et al. [61]. Both results are again confirmed by the results of our study since all the experimental animals in the highest tested group of 20,000 µg/L PCZ died/the mortality achieved 100%. Domingues et al. [61] also reported delayed hatching and various developmental malformations such as spine malformation or edemas. However, this was observed at concentrations higher than 1200 µg/L, which is higher than our highest survival group (a concentration of 200 µg/L). In the literature, MTCH was also reported to affect growth. In particular, crayfish (*Astacus* sp.) exposed to MTCH (0.0115 µmol/L and 0.0790 µmol/L) and metazachlor oxalamic acid (0.0117 µmol/L and 0.0805 µmol/L) showed lower growth in early-life stages compared to the control [11].

MCPA is believed to demonstrate low to moderate toxicity to fish and aquatic plants. The LC50 values for fishes vary among scientific studies, as values between 50 and 100 mg/L were reported [62,63]. In our study, specimens of *X. laevis* exhibited 100% mortality in the highest concentration group. Regarding both fish species, exposure to 100,000 µg/L MCPA resulted in elevated malformation rate (heart edemas, blood clots). Similarly, Sun et al. [64] reported an increased incidence of malformations on *C. carpio*, such as pericardial edema, body malformations, and spinal curvatures together with delayed hatching, after exposure to concentrations 52 mg/L of MCPA-Na after 96hpf.

To sum up the results of our study, *X. laevis* seems to be the most sensitive model organism used in this study towards the pesticides tested. Among the substances, PCZ showed the highest toxicity for the species tested. Addressing sublethal effects, the most common malformations observed were heart edemas and blood clots in *D. rerio* and *C. carpio* embryos exposed to 100,000 µg/L MCPA and delayed hatching of *C. carpio* exposed to several sublethal concentrations of MTCH, PCZ, and MCPA.

#### 3.1.2. Effects of Selected Parabens

During these 96 h tests, mortality, the occurrence of malformations, and hatching rates of *D. rerio*, *C. carpio*, and *X. laevis* embryos after exposure to MTP, PPP, and BTP were observed.

Zebrafish (*D. rerio*)

Regarding mortality, PPP and BTP seem to be more toxic in comparison to MTP. Both PPP and BTP caused 100% mortality of *D. rerio* embryos at the concentration of 100,000 µg/L (*p* < 0.01), whereas MTP caused 33.3% mortality in this concentration (*p* < 0.05). The most common malformations observed in the experiments were heart edemas and blood clots. They were observed in the group exposed to MTZ at 100,000 µg/L and BTP at 1000 and 100,000 µg/L. Exposure to PPP did not result in any developmental malformations. At 72 hpf, both MTP and BTP significantly delayed hatching of embryos in comparison to the control among the exposed groups, with the exception of the lowest one and also the highest one, where 100% mortality was reported. PPP delayed hatching only in the group exposed to 1000 µg/L. (The highest concentration also had 100% mortality.)

Common carp (*C. carpio*)

All three tested parabens caused 100% mortality at the highest tested concentrations. No effects on the occurrence of developmental malformations were observed among groups. Concerning the speed of hatching, it was delayed at 72 hpf after exposure to MTP at 50, 500, and 5000 µg/L. Furthermore, it was delayed after exposure to 1000 µg/L PPP.

African clawed frog (*X. laevis*)

Exposure to MTP, PPP, and BTP resulted in 100% mortality in the highest tested concentrations and in elevated mortality in the group exposed to 5000 µg/L MTP and 1000 µg/L BTP. Hatching was delayed in all groups exposed to BTP and also in groups exposed to 5000 µg/L MTP. Higher malformation occurrence was reported in group exposed to 1000 µg/L BTP, while total body deformation and heart edemas were the most frequently described malformations.

To summarize the results of lethal and sublethal endpoint monitoring during the embryonal toxicity tests, all three parabens exhibited acute toxicity toward fish and amphibian embryos. While the highest tested concentration of 100,000 µg/L of BTP and PPP caused 100% mortality among tested species, in the case of 100,000 µg/L MTP, 100% mortality was observed only in *C. carpio* and *X. laevis* experiments. Anyway, even exposure to 100,000 µg/L MTP resulted in a significant increase in *D. rerio* mortality in this group (33.3%). Dambal et al. [33] estimated the 96LC50 of MTP to be 65,000 µg/L on *D. rerio*. Merola et al. [65] observed significant mortality (more than 60%, *p* < 0.05) at the concentration 80,000 µg/L of MTP in *D. rerio* embryos. Bereketoglu [66] observed 100% mortality after exposure to 10 µM PPP on *D. rerio*. Merola et al. [67] exposed *D. rerio* to concentrations 0.25, 0.5, 1, 2.5, and 5 mg/L of BTP to for a period of 4 days. As a result, the LC50 was determined to be 2.3 mg/L. Also, embryos who were exposed to the highest concentrations (2.5 and 5 mg/L) of BTP showed a significant decrease in hatching rate (*p* < 0.05) which was 10–30%.

Regarding sublethal endpoints, delayed hatching was observed among species in several groups exposed to parabens. Similar effects were described in the literature. Merola et al. [65] reported a significant reduction in the hatching rate at concentrations of 30,000, 60,000, and 80,000 µg/L of MTP. Similarly, Bereketoglu et al. [66] described delayed hatching after exposure to MTP and PPP. They also observed in 10 μM PPP that the embryos showed less mobility after hatching compared to the 1 μM propylparaben and the control group. Gonzáles-Doncel [68] exposed Japanese medaka (*Oryzias latipes*) for 13 days to 40, 400, 1000, and 4000 μg/L PPP. At 244 hpf, a reduced developmental rate was observed at 4000 μg/L. Kang et al. [69] determined the LC50 for copepod *Tigriopus japonicus* males and females to be 114 and 357 μg/L, respectively.

Additionally, various malformations related to the cardiovascular system (such as heart edemas, blood stasis, and blood cloths) after exposure to parabens were described in our study as well as some other studies [33,65].

### 3.2. Embryonal Toxicity Tests—Study on Gene Expression

#### 3.2.1. Effects of Selected Pesticides

**Figure 1:**
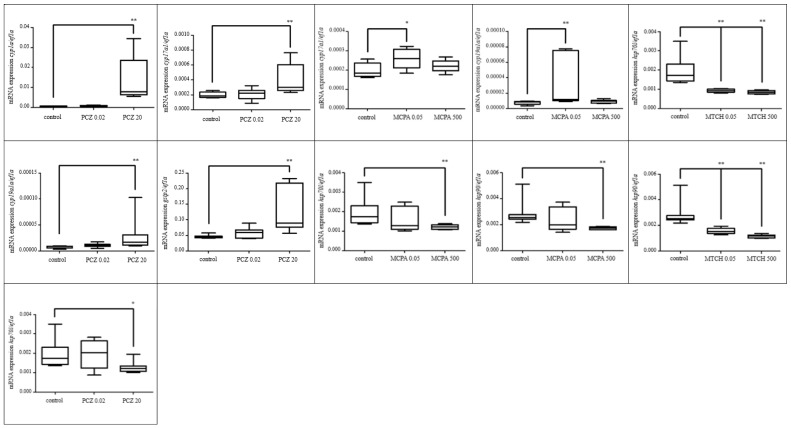
Relative mRNA expression of selected genes in *D. rerio* embryos exposed to various pesticides. Concentrations are indicated in µg/L. Box plot graphs were constructed using median, Q1, Q2 with whiskers of maximum 1.5 interquartile range. Statistically significant differences are indicated by * (*p* < 0.05) or ** (*p* < 0.01).

**Figure 2:**
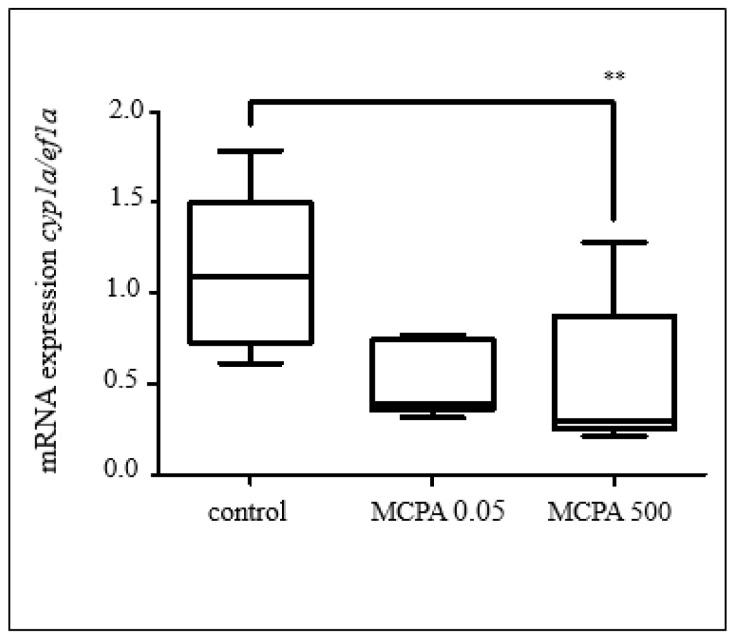
Relative mRNA expression of selected genes in *C. carpio* embryos exposed to MCPA. Concentrations are indicated in µg/L. Box plot graphs were constructed using median, Q1, and Q2 with whiskers of a maximum 1.5 interquartile range. Statistically significant differences are indicated by ** (*p* < 0.01).

**Figure 3:**
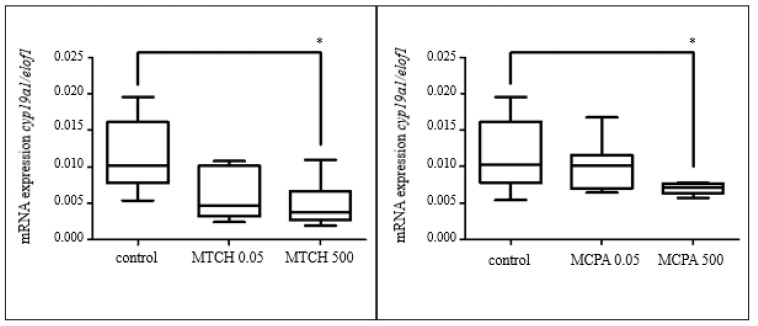
Relative mRNA expression of selected genes in *X. tropicalis* embryos exposed to various pesticides. Concentrations are indicated in µg/L. Box plot graphs were constructed using median, Q1, and Q2 with whiskers of a maximum 1.5 interquartile range. Statistically significant differences are indicated by * (*p* < 0.05).

During these 96 h tests, embryos of *D. rerio*, *C. carpio*, and *X. laevis* were exposed to MTCH, PCZ, and MCPA at selected concentrations indicated in Table 1 (only values indicated in bold) and followingly sampled and handled as indicated in Section 2.4. Relative mRNA expression of genes indicated in Table 1 was assessed. Figure 1, Figure 2 and Figure 3 are showing genes where significant changes in up/down-regulation were reported; otherwise, non-significant data are not reported.

PCZ was found to significantly affect the regulation of mRNA expression in the majority of analyzed genes in *D. rerio* (Figure 1). The effects of PCZ on gene expression have been also addressed in other scientific studies. [70] exposed zebrafish larvae (*D. rerio*), to a series of concentrations (0, 20, 100, and 500 μg/L) for a period of 7 days. The transcriptome analysis of differentially expressed genes revealed that PCZ disturbed glycolipid metabolism and amino acid metabolism and could induce oxidative stress. In addition, carnitine, acyl-carnitine, and amino acid metabolites were affected by PCZ. Experiments carried out by [70] confirmed that PCZ exposure elevated the level of triglycerides, decreased glucose content, and changed the expression levels of key genes associated with glycolipid metabolism (tested the content of the pesticide PCZ).

In our study, the genes selected for *D. rerio* toxicity tests play either a prominent role in detoxification and sex hormone production or indicate cell stress. Cytochrome P450 family 1 subfamily A member (in this study represented by *cyp1a* gene) belongs to one of the most important group of enzymes needed for the bioactivation of xenobiotics with a broad spectrum of substrates, including drugs and environmental pollutants [71,72]. Its expression was significantly up-regulated (*p* < 0.01) after exposure to 20 µg/L PCZ. Similarly, glutathione-*S*-transferase (GST) represents a family of phase II enzymes that detoxify a wide range of toxicants and reactive intermediates. GST functions by conjugating electrophilic substrates, both endogenous and exogenous, to reduced glutathione. The conjugates are generally less toxic than the unconjugated metabolite, more water-soluble, and thus, more easily excreted from the cell [73]. In this study, GST type p2 (evaluated through gene *gstp2*) expression was up-regulated (*p* < 0.01) in *D. rerio* embryos after exposure to 20 µg/L PCZ. In the study of Domingues et al. [61], *D. rerio* larvae exposed to 0.3 mg/L PCZ for 144 h increased GST activity. They also refer to a previous study carried out by Sanchez et al. [74], pointing out that PCZ is known for being able to modulate not only the phase II biotransformation enzymatic activities such as GST but also cytochrome P450 enzymes. Neither MCPA nor MTCH affected the expression of *cyp1a* in *D. rerio*. Contrary, in *C. carpio*, exposure to 500 µg/L MCPA resulted in *cyp1a* down-regulation (*p* < 0.01; Figure 2). Even though PCZ did not have any reported effects on gene expression in C. carpio, according to literature, moderate DNA damage induction was observed in crucian carp (*Carassius carassius*) after 4 days long exposure to 1.29 μg/L MTCH [12]

PCZ, like other imidazole fungicides, can strongly interact with the iron atom of CYP [75]. PCZ together with other imidazoles inhibits the activity of other enzymes in the cytochrome P450 family, such as CYP19, i.e., aromatase. Therefore, PCZ is also believed to be an endocrine disruptor that inhibits aromatase [70]. Similarly, MCPA was reported to have a stimulatory effect on steroidogenesis in a study made by Orton et al. [76].

Cytochrome P450 17 A 1 (encoded by *cyp17a1* gene) is an important enzyme in steroid hormone synthesis. Aromatase A (encoded by the *cyp19a1a* gene) catalyzes the conversion of androgens into estrogens [77]. In our study, expression of both of these genes in *D. rerio* was up-regulated (*p* < 0.01) at 20 µg/L PCZ but also 0.05 µg/L MCPA (*p* < 0.05). Contrary, in *X. laevis*, *cyp19a1a* was downregulated at 500 µg/L MCPA (*p* < 0.01; Figure 3). The same gene was down-regulated in *X. laevis* even after exposure to MTCH at 500 µg/L (*p* < 0.05).

At this point, it is necessary to emphasize that it is incorrect to assume that responses at the mRNA level reflect the response at the protein level or at the level of active enzyme, i.e., that there is a statistical correlation between levels of mRNA, protein, and active enzyme [78]. Therefore, an up-regulation of the cytochrome P450 family genes does not exclude the possible inhibition of related enzyme activity. Contrary, it supports the hypothesis that various pesticides affect the normal endocrine functions in exposed organisms. By up-regulation, a cell increases its response to a substance from outside to carry out a specific function. Contrarily, gene expression might also be downregulated after exposure to various chemicals suggesting too long or too intense inducement might result in cell exhaustion.

Heat shock proteins consist of several families of highly conserved proteins whose expression is upregulated in response to a broad range of environmental stressors [79]. However, some of them have also been proven to be essentially involved in embryonic development. For example, gene *hsp70l* is required for the formation of the zebrafish and other vertebrate lenses [80]. Gene *hsp90* is required for the normal differentiation of somatic muscle pioneer cells [81]. From Figure 1 it is obvious that all three tested substances (PCZ, *p* < 0.05; MTCH, *p* < 0.01; MCPA, *p* < 0.01) had a significant effect on *hsp70l* and *hsp90* expression in *D. rerio* embryos. In case of MTCH, even the lowest, environmentally relevant concentration of 0.05 µg/L was proven to affect both of these genes (*p* < 0.01).

#### 3.2.2. Effect of Selected Parabens

**Figure 4:**
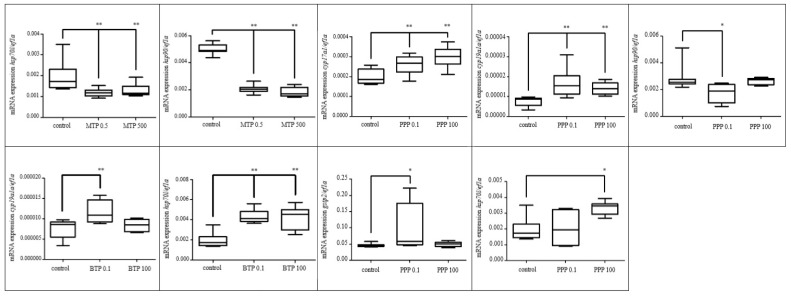
Relative mRNA expression of selected genes in *D. rerio* embryos exposed to various parabens. Concentrations are indicated in µg/L. Box plot graphs were constructed using median, Q1, and Q2 with whiskers of a maximum 1.5 interquartile range. Statistically significant differences are indicated by * (*p <* 0.05) or ** (*p* < 0.01).

In *D. rerio*, exposure to MTP resulted in the down-regulation of both *hsp70l* and *hsp90* in both tested concentrations (*p* < 0.01, Figure 4). PPP caused down-regulation of *hsp90* at 0.1 µg/L. Contrary, exposure to BTP caused up-regulation of *hsp70l* (*p* < 0.01). PPP caused up-regulation (*p* < 0.05) of *hsp70l* in the group exposed to 100 µg/L. The expression of *gst p2* was upregulated (*p* < 0.05) after exposure to 0.1 µg/L PPP. Significant up-regulation of both *cyp17a1* and *cyp19a1a* expression followed exposure to 0.1 and 100 µg/L PPP (*p* < 0.01), while in BTP only *cyp19a1a* was upregulated at 0.1 µg/L (*p* < 0.01).

**Figure 5:**
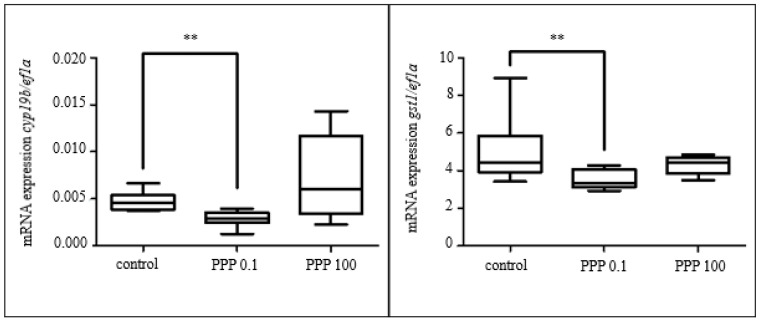
Relative mRNA expression of selected genes in *C. carpio* embryos exposed to PPP. Concentrations are indicated in µg/L. Box plot graphs were constructed using median, Q1, and Q2 with whiskers of a maximum 1.5 interquartile range. Statistically significant differences are indicated by ** (*p* < 0.01).

In *C. carpio*, our study showed a statistically significant decrease (*p* < 0.01) in *cyp19b* and *gst1* in the lower tested concentration of PPP. The results are shown in Figure 5.

**Figure 6:**
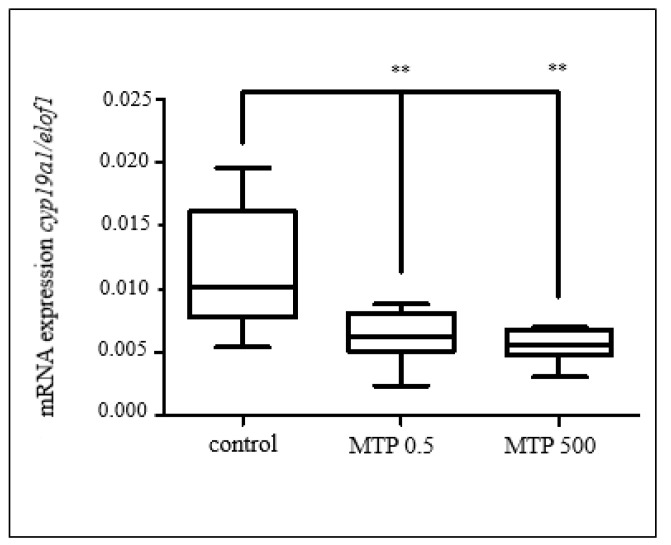
Relative mRNA expression of selected genes in *X. laevis* embryos exposed to MTP. Concentrations are indicated in µg/L. Box plot graphs were constructed using median, Q1, Q2 with whiskers of maximum 1.5 interquartile range. Statistically significant differences are indicated ** (*p* < 0.01).

Significant down-regulation of *cyp19a1* (*p* < 0.01) was found after exposure to both concentrations of MTP in *X. laevis* embryos. The results are shown in Figure 6.

To sum up the above-described results up, MTP, PPP, and BTP had a significant effect on *cyp19* (and for *D. rerio* even *cyp17a1* in PPP) expression among the species tested (even though none of the chemicals affected all three species). Also, in the experiment with *D. rerio*, both *hsp70l* and *hsp90* expressions were affected after exposure to MTP, PPP, and BTP even in the lower, environmentally relevant, concentration (with exception in BTP 0.1 µg/L, where no changes occurred). PPP even affected the expression of GST-related genes in *D. rerio* and *C. carpio* experiments.

### 3.3. Embryo-Larval Toxicity Test

#### 3.3.1. Mortality

At 31 dpf, significantly increased mortality (60.7%) was found at the highest tested concentration of 1000 µg/L PCZ. As described in Section 3.1.1, PCZ 96hLC50 for fish varies in literature with values between 1.5–8.5 mg/L. However, as evident from the result of these subchronic toxicity tests, even the concentrations of 1 mg/L have a tremendous effect on the early-life stages of fish when exposed for a prolonged time.

#### 3.3.2. Hatching, Development, and Morphometric and Condition Characteristics

Hatching started at the third dpf. At 4 dpf, PCZ significantly reduced the hatching rate at all tested concentrations in comparison to the control groups. Hatching was completed by 5 dpf, when all the embryos throughout the experiment were reportedly hatched. As indicated already in the Materials and Methods section, specimens were taken on dpf 6, 13, 20, 27, and 31 to determine their developmental stage and various morphometric and condition characteristics. Based on the data obtained, at 6 dpf, the development was significantly delayed in all groups exposed to PCZ. Moreover, the total length of the body was significantly shorter in these groups in comparison to the control. This effect was probably the consequence of the delayed hatching described above. As the experiment went on, the differences between the control and exposed groups evened out.

#### 3.3.3. Histopathological Examination

During the histological examination, several pathological changes were detected in the brain, eyes, skin, and gills. In the literature, Haselman et al. [13] tested PCZ on *X. laevis* and described moderate hepatocellular degeneration with cytoplasmatic inclusion in *X. laevis* liver after exposure to 180 µg/L PCZ for a period of two months. During the test on *C. carpio,* we observed dystrophy of liver parenchyma in both control groups and all tested concentrations, but in higher concentrations (100 and 1000 µg/L), these changes were more progressive (all-over dystrophy of liver parenchyma).

**Figure 7:**
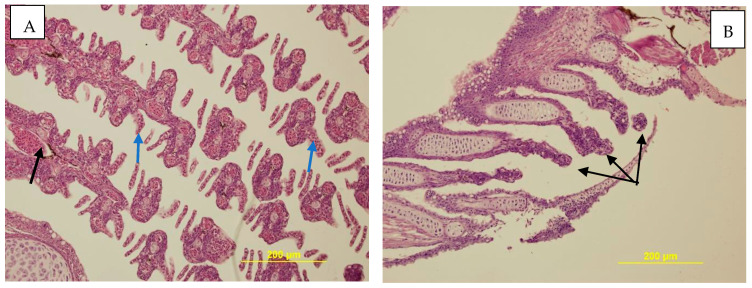
The gills at the control group (**A**); (black arrow vessel tinting, blue arrow leaf deformation), and gills at the concentration 1000 µg/L of PCZ (**B**); (black arrows showing total deformation of gill leaves).

On the surface of the skin of individuals from both control groups and individuals from 0.1 µg/L, sporadic mucus cells were observed. However, in *C. carpio* exposed to 1, 10, and 100 µg/L of PCZ, significantly more mucus cells on the skin were observed. Moreover, at the highest tested concentration, strong surface alteration of the skin was observed. Additionally, a deformation of gill leaves was observed in the control groups and at the concentrations 0.1, 1, 10, and 100 µg/L PCZ. However, the total absence of gill leaves was reported at the concentration of 1000 µg/L (Figure 7). Some studies show that if oxidative stress occurs in fish exposed to some pesticides, then histopathological changes are observed. Yang et al. [82] described that reactive oxygen species (ROS) induced histopathological changes in the liver, gills, and brain after exposure to 2 µg/L deltamethrin in silver carp (*Hypophthalmichthys monolithic*). Deltamethrin inducted a marker of inflammation and oxidative stress, which caused histopathological alteration in the gill, liver, and brain of carp [82].

**Figure 8:**
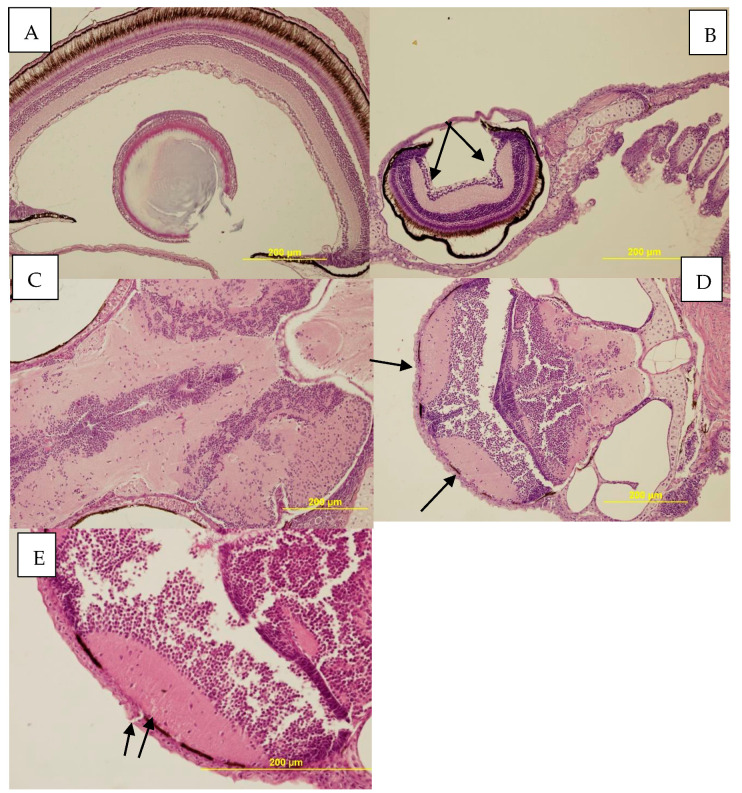
The eye in the control group (**A**) and deformation of an eye after exposure to 1000 µg/L of PCZ (**B**), indicated by black arrows. The normal structure of the brain at the control group (**C**) and (submeningeal lesion with alterations in the brain) after exposure to 1000 µg/L of PCZ; (**D**) magnification 200×; (**E**) magnification 400×.

Additionally, in our study, the highest tested concentration of 1000 µg/L was found to cause deformation of the eye and brain. In individuals exposed to 1000 µg/L PCZ, significant shape deformation of the eyes and submeningeal lesion with alterations in the brain were observed (Figure 8).

Several studies in which different kinds of pesticides were tested showed that pesticides have the potential to cause histopathological changes in fish. Sepici-Dicel et al. [83] tested 10 µg/L of cyfluthrin on *C. carpio* for 48 hours, and this pesticide caused hyperemia in the brain, hydropic degeneration of the liver, and telangiectasis in gills. In addition, insecticide deltamethrin (2 µg/L, after 96 h of exposure) caused histopathological changes in the brain such as hemorrhage, spongiosis, and neuronal degeneration of *H. molitric* [84].

#### 3.3.4. Indices on Oxidative Damage

**Figure 9:**
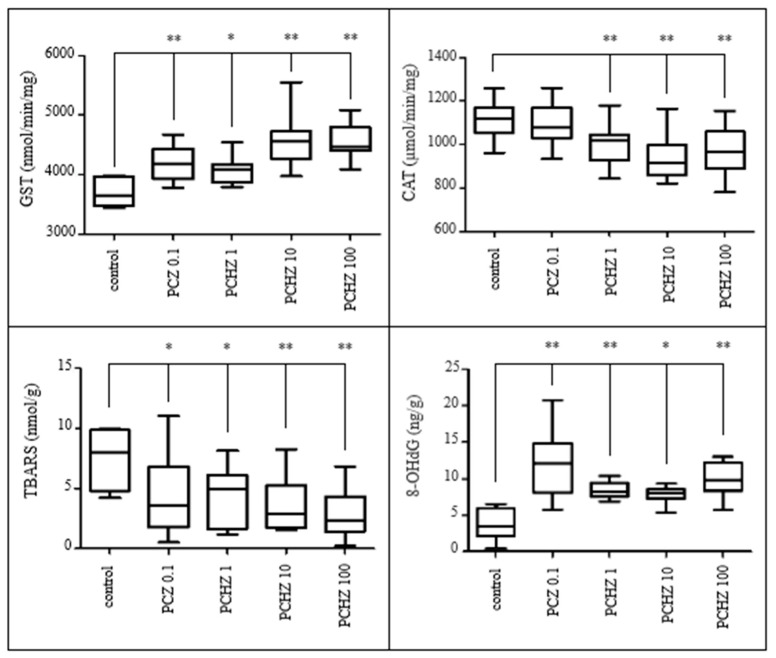
Results of various oxidative stress indices in *C. carpio* larvae after exposure to PCZ (expressed to mg or g tissue). Concentrations are indicated in µg/L. Box plot graphs were constructed using median, Q1, and Q2 with whiskers of a maximum 1.5 interquartile range. Statistically significant differences are indicated by * (*p <* 0.05) or ** (*p* < 0.01).

Water pollution caused by pesticides is a major contributor to the imbalance in ROS production and antioxidant systems damage in non-target organisms [85]. The most important enzymes regulating ROS are SOD and CAT. An additional key antioxidant enzyme providing cellular defense against ROS is GST [86]. To reveal the potential of PCZ to cause oxidative stress and, consequently, oxidative damage to the organism, several indices were analyzed in this part of the study, namely SOD, CAT, and GST activity, and also TBARS and oxidative damage to proteins and DNA assessment. Unfortunately, due to high mortality, not enough material was collected to include the highest tested concentration of 1000 µg/L PCZ in this assessment. As indicated in Figure 9, both activities of CAT as well as TBARS content decreased in a concentration-dependent manner (*p* < 0.01; *p* < 0.05). Contrarily, GST activity and 8-OHdG content increased after exposure to PCZ (*p* < 0.05; *p* < 0.05).

Modra et al. [87] studied the effects of subchronic exposure to prochloraz at concentrations of 0.05, 0.15, and 0.38 mg/L (through exposure to the pesticide Spartakus) on juvenile *C. carpio* for 28 days. They found GST activity to be induced by all concentrations tested.

In the scientific literature, PCZ was reported to cause oxidative stress and related oxidative damage as well as have genotoxic properties [88]. Accordingly, elevated 8-OHdG values, a critical biomarker of oxidative damage to nuclear and mitochondrial DNA, were found among the groups including the lowest tested concentration of 0.1 µg/L PCZ (*p* < 0.01) in our study (after 31 days).

In our test, the value of TBARS significantly decreased in all treated groups. These results suggest that the capacity of the antioxidant system was probably not exhausted after exposure to tested substances. Lidova et al. [89] also observed a significant decrease in TBARS value in juvenile marbled crayfish after 72 h exposure to 0.02 μg/L and 0.05 μg/L of Cyperkill 25 EC (containing 250 g/L cypermethrin). Stara et al. [90] found a decrease in TBARS value in common carp brains exposed to Cyperkill 25 EC (45.7 μg/L). On the contrary, Toni et al. [91] found a significant increase in TBARS levels in the liver, brain, and muscle of common carp exposed to varying concentrations of tebuconazole. In our study, the changed antioxidant activities (GST and CAT) in exposed common carp were related to the detoxification of oxidative stress, suggesting that the tested substances affect antioxidant defense systems. Acute exposure of common carp to Cyperkill 25 EC (4.57 and 45.7 μg/L) leads to a decrease in muscle and brain CAT activity [90].

The PCZ-exposed carp larvae showed significant changes in SOD, CAT, GST activity, and 8-OHdG content, apparently maintaining cells in the equilibrium state and combating ROS production since this was an observed decrease of oxidative damage such as lipoperoxidation. The changes in antioxidant levels in exposed larvae carp were related to the detoxification of oxidative stress, suggesting that the tested substances affect antioxidant defense systems.

#### 3.3.5. Gene Expression

Regarding the results obtained from qPCR, significant upregulation of *cyp1a* expression (*p* < 0.01) was observed after exposure to 100 and 1000 µg/L PCZ. This result is confirmed by results obtained in Section 3.2.1, where *cyp1a* expression was also upregulated after exposure to 20 µg/L PCZ in *D. rerio* embryos (but not *C. carpio* embryos, which seem to be less sensitive and are affected by higher concentrations).

**Figure 10:**
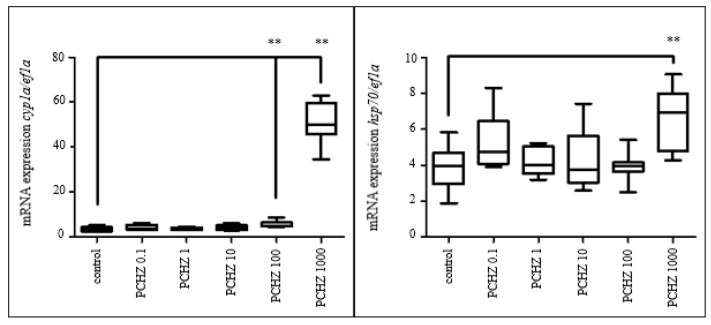
Relative mRNA expression of selected genes in *C. carpio* larvae exposed to PCZ. Concentrations are indicated in µg/L. Box plot graphs were constructed using median, Q1, and Q2 with whiskers of a maximum 1.5 interquartile range. Statistically significant differences are indicated by ** (*p* < 0.01).

Further, significant up-regulation of *hsp70* expression (*p* < 0.01) was observed at 1000 µg/L PCZ in this embryo-larval test. The results are shown in Figure 10.

## 4. Conclusions

This extensive study was designed to help to elucidate the effects of widely used pesticides and parabens on aquatic non-target biota such as fish and amphibians using a wide range of lethal and sublethal endpoints. Both acute embryonic and subchronic embryo-larval toxicity tests were carried out to understand not only the acute toxicity but also the long-term effects of chemicals, which is more environmentally realistic.

The qPCR analysis surprisingly showed that even low concentrations have the potential to affect the expression of various genes playing roles in detoxification and sex hormone production, or they indicated cell stress in the early-life stage of organisms after only short-term exposure (4 days). Additionally, the results from subchronic toxicity tests with *C. carpio* larvae on PCZ show that this substance is highly toxic, causing histopathological changes and oxidative damage.

## Figures and Tables

**Table 1:** Chemicals tested in the study, their concentrations, and an indication of the solvent used.

Group Tested	Concentrations Tested (µg/L)	The Concentration of Solvent Used in Testing Solutions 3
**Control**		**X**
**Control with ethanol**		**Ethanol 0.01%**
**Control with DMSO**		**DMSO 0.01%**
**MTCH**	**0.05**; 50; **500**; 5000;100,000	DMSO 0.01%
**PCZ**	**0.02**; 0.2; **20**; 200; 20,000	DMSO 0.01%
**MCPA**	**0.05**; 50; **500**; 5000; 100,000	Ethanol 0.01%
**MTP**	**0.5**; 50; **500**; 5000; 100,000	Ethanol 0.01%
**PPP**	**0.1**; 10; **100**; 1000; 100,000	Ethanol 0.01%
**BTP**	**0.1**; 10; **100**; 1000; 100,000	Ethanol 0.01%

## Data Availability

Not applicable.

## Supplementary Materials

The following supporting information can be downloaded at: https://www.mdpi.com/article/10.3390/toxics11040333/s1, Table S1: Information on primers used. References [92,93,94,95] are cited in the supplementary materials.
